# Multiple and mixed methods in formative evaluation: Is more better? Reflections from a South African study

**DOI:** 10.1186/s12874-016-0273-5

**Published:** 2016-12-15

**Authors:** Willem Odendaal, Salla Atkins, Simon Lewin

**Affiliations:** 1 Health Systems Research Unit, Medical Research Council of South Africa, Cape Town, South Africa; 2 Department of Public Health Sciences, Karolinska Institutet, Stockholm, Sweden; 3 School of Health Sciences, University of Tampere, Tampere, Finland; 4 Global Health Unit, Norwegian Institute of Public Health, Oslo, Norway

**Keywords:** Mixed methods, Multiple methods, Formative evaluation, Complex interventions, Community-based programmes, Diary-keeping, Observations, Time-and-motion, Survey, Lay health workers

## Abstract

**Background:**

Formative programme evaluations assess intervention implementation processes, and are seen widely as a way of unlocking the ‘black box’ of any programme in order to explore and understand why a programme functions as it does. However, few critical assessments of the methods used in such evaluations are available, and there are especially few that reflect on how well the evaluation achieved its objectives. This paper describes a formative evaluation of a community-based lay health worker programme for TB and HIV/AIDS clients across three low-income communities in South Africa. It assesses each of the methods used in relation to the evaluation objectives, and offers suggestions on ways of optimising the use of multiple, mixed-methods within formative evaluations of complex health system interventions.

**Methods:**

The evaluation’s qualitative methods comprised interviews, focus groups, observations and diary keeping. Quantitative methods included a time-and-motion study of the lay health workers’ scope of practice and a client survey. The authors conceptualised and conducted the evaluation, and through iterative discussions, assessed the methods used and their results.

**Results:**

Overall, the evaluation highlighted programme issues and insights beyond the reach of traditional single methods evaluations. The strengths of the multiple, mixed-methods in this evaluation included a detailed description and nuanced understanding of the programme and its implementation, and triangulation of the perspectives and experiences of clients, lay health workers, and programme managers. However, the use of multiple methods needs to be carefully planned and implemented as this approach can overstretch the logistic and analytic resources of an evaluation.

**Conclusions:**

For complex interventions, formative evaluation designs including multiple qualitative and quantitative methods hold distinct advantages over single method evaluations. However, their value is not in the number of methods used, but in how each method matches the evaluation questions and the scientific integrity with which the methods are selected and implemented.

## Background

Evidence of the effectiveness of healthcare programmes is important to informing policy and practice decisions [1–3]. Understanding how these programmes are implemented, including how contextual and programmatic factors contribute to the success or failure of a programme [4], is equally important [5]. Formative evaluations – evaluations that assess intervention implementation processes [6] – can contribute to this understanding. These evaluations should aim to identify the key components of a programme as implemented and to explore the factors affecting its implementation, in relation to the implementation context. Formative evaluations findings can guide the effective replication of programmes in other settings [7]. Such formative evaluations are often designed as multiple, mixed-methods evaluations [8], defined as the application of a combination of qualitative and quantitative approaches [9–11], to allow them to address adequately a wide range of questions regarding how a programme was implemented.

Multiple, mixed-methods evaluations are used widely, particularly for programmes that involve complex interventions or a complex mix of interventions [12, 13]. Multiple, mixed-methods approaches have a number of advantages, including providing a more holistic and textured picture of a programme and its implementation [12, 14, 15], and having the potential to redress the inherent biases that any single method has [16, 17]. They also allow the corroboration, or triangulation, of findings [11, 16, 18, 19]. However, these approaches raise a number of challenges including assembling a research team with skills and experience across multiple methods [20]; acquiring the resources needed to implement data collection using multiple strategies [21]; and undertaking analysis of data collected using multiple approaches [22].

This paper discusses the strengths and weaknesses of a multiple, mixed-methods approach applied in a formative evaluation of a community-based health programme that addressed the burden of HIV/AIDS and tuberculosis (TB) in Cape Town, South Africa. This evaluation, the findings of which are reported elsewhere [23], comprised an unusually wide range of methods, including two methods – time-and-motion analysis and diary keeping – that are less commonly reported in the context of programme evaluation. The evaluation therefore provided a valuable opportunity to reflect on how we applied these methods and the implications of the methodological choices that we made. This paper aims to assess each of the methods used in relation to the evaluation objectives, and offers suggestions regarding ways of optimising the use of multiple, mixed-methods within formative evaluations of complex interventions.

## Methods

### Methods used to develop this paper

This paper is based on critical reflections by the research team on the evaluation we conducted. Both during and after the evaluation, the evaluation team reflected on the methods used, paying attention to the following issues: (i) ensuring that all programme components were evaluated; (ii) the comparative ease or difficulty in implementing data collection; (iii) the perceived contribution of the findings from each method to the overall evaluation findings; and (iv) the process of drawing together the findings from across the study. The lead researcher (WO) kept notes of these discussions, which occurred mainly during progress reports to the evaluation team, and also kept process notes of methodological issues that arose during the fieldwork. These notes, together with the reflexive discussions within the evaluation team, formed the basis for this paper. The ideas described here evolved further through the writing process.

### The evaluation: setting and programme description

TB and HIV/AIDS remain high priority public health problems in South Africa: at the time of the evaluation presented here (2009–2010), South Africa had the largest HIV/AIDS epidemic in the world, with approximately 5.6 million people living with HIV/AIDS [24]. The country also had the third highest TB burden in the world, with an infection rate of 981/100 000 [25]. This situation was complicated by the high rates of TB/HIV co-infection: 128,457 people were reported to be co-infected with TB and HIV in 2010 in South Africa [26]. At the time of this study, the Cape Town Metropole (Metro), the study site, had the highest TB incidence rate in South Africa of 752 per 100 000 population [27]. The antenatal HIV prevalence in this health district was 19.1% (range 8.8% – 33.1%). This was lower than the national prevalence of 30.2% [27]. Approximately 47% of TB clients in this district were also living with HIV/AIDS [28].

The Metro district has been active in developing community-based programmes to deliver care to people living with TB and HIV/AIDS. The district has a strong focus on employing lay health workers (LHWs – sometimes referred to as community health workers) as treatment providers in these programmes [29], and of integrating care for co-infected clients [30]. Within the Metro, a number of different models for delivering TB and HIV/AIDS care have been tested, including (i) the Enhanced TB Adherence (ETA) programme, where TB treatment was modeled on the anti-retroviral therapy (ART) programme’s supported self-administration approach [30]; and (ii) a model of care delivered with a mix of directly-observed-therapy (DOT) and ETA for TB clients and tailored support for ART clients [23]. These models were supported by the two health authorities in the Metro: the City of Cape Town, Department of Health (CDoH) and the Western Cape Provincial Government Department of Health (WCDoH), in collaboration with a number of non-governmental organisations (NGOs) who employed and managed the LHWs involved in these programmes.

The formative evaluation that provided the data for this methodological assessment was commissioned by an NGO who provided community-based primary healthcare services in poorer areas of the Metro, on behalf of the Metro health authorities. This programme of community-based services used different models across the programme sites to integrate LHW-led care for people with TB and/or HIV/AIDS (Table 1). However, these integration models used the same LHW structure across the sites. Specifically, each LHW team comprised of a group of LHWs and a team leader. The team leaders were, like the LHWs, recruited from the community, and both groups had no formal, professionally certified healthcare training but received training in the context of their work. The team leaders had been promoted, based on their experience and quality of work, to manage a LHW team. They had less contact with clients than LHWs, and served as link between the staff of the local primary health care clinic and LHWs in the field. The LHWs conducted treatment and adherence support visits to clients. These visits included observation of the taking of TB treatment and ART, pill counts, checking for treatment side-effects and social support to clients. The three LHW teams were all female.

**Table 1:** Different models of care

Site 1	Site 2	Site 3
Clients on TB treatment received directly observed therapy with a LHW for the first month of treatment. Thereafter, treatment was self-administered with LHW support visits. LHWs monitored ART clients daily for the first two weeks of treatment. For the next two weeks, stable clients were visited weekly and thereafter they received one or two visits per month. Clients doing not well on ART were more closely monitored and often referred back to the clinic.	TB treatment and ART followed the same strategy: after two weeks of clinic-based treatment, monthly medication was supplied to clients who were assessed as being adherent to treatment. These clients then self-administered their treatment with weekly LHW support visits.	DOT was administered to all TB clients. The ART protocol was the same as in Site 1.

The NGO purposefully selected three low-income communities for the study, based on the different models of care implemented in these sites. All three sites had ethnically diverse populations and high levels of unemployment, with a significant proportion of residents below the government’s poverty threshold of USD 320 per month (Table 2). Most residents lived in informal housing. Site 3 differed in that it was a slightly wealthier community that included formal housing areas.

**Table 2:** Demographic profiles of the selected study communities [46–48]

Site	Male	Unemployment rate	Income (<320 USD per month)
1	55%	33%	79%
2	48%	45%	74%
3	49%	27%	50%

### The evaluation methods

#### Aim of the evaluation

The evaluation aimed to explore the strengths and weaknesses of the community-based treatment support programme in order to inform programme development. The objectives were, firstly, to provide a detailed description of the programme and its implementation; and, secondly, to explore the experiences and perceptions of clients, LHWs and programme managers regarding the programme. The evaluation did not aim to assess the effectiveness of the programme in relation to client health outcomes.

#### Evaluation design: five sub-studies

The evaluation objectives led to the mixed-methods approach summarised in Table 3. Each method, which we refer to as a ‘sub-study’, targeted different actors and components of the programme. Our approach was informed by a number of considerations: it not only matched the complexity of the programme, but offered the required depth (through the use of qualitative approaches) and breadth (through the use of a quantitative survey approach) of information sought by the commissioning NGO. Table 3 outlines the rationale for and implementation of each method.

**Table 3:** Evaluation design

Objectives	Methodological approach used in the sub-study that addressed each objective (study sites in which the sub-study was conducted)	Rationale for the methods chosen	Implementation and data analysis
*Objective 1* To describe the programme and its implementation	*Sub-study 1: Time-and-motion*, in which the LHWs’ activities and time spent on each activity were recorded. (Sites 1–3)	1) To develop a detailed description of LHW activities, including any variation between sites and to gain insight into how LHWs organized their work 2) To understand how the programme was implemented to help better understand the contextual issues impacting on the programme	1) Each LHW was shadowed for one day by an evaluator with a stop-watch and a checklist for recording time per activity 2) Data were categorised into client-contact, walking between houses, and waiting time 3) Descriptive analysis in Excel was performed
	*Sub-study 3: Structured observations* of the content and nature of the interactions and communication between LHWs and clients during the LHW visits. (Site 1)	To obtain in-depth information about how the LHWs provide treatment and adherence support in the field	1) LHWs with whom we had good rapport were asked to identify adherent and non-adherent clients 2) An evaluator used an observation guide to record (i) interactions between LHW and client; (ii) mood during the visit; (iii) content and pattern of the conversation; and (iv) any additional issues that impacted on the visit 3) The visit was audio-recorded 4) The recordings were transcribed and analysed thematically
*Objective 2* To solicit the experiences and perceptions about the programme from clients, lay health workers, and programme managers	*Sub-study 2: Anonymous client survey* (Sites 1–3)	To gain information about clients’ experiences and assessments of the programme	1) The questionnaire was administered in the three main languages of the respective sites 2) Clinic staff distributed the information letters to all TB, HIV/AIDS and co-infected clients as they attended the clinic, and referred those interested in participating to the evaluator, who administered the survey at the clinic 3) Descriptive analysis in Excel was performed
	*Sub-study 4: Interviews* with clients, LHWs, NGO staff and managers within the relevant health authorities (Sites 1–3)	To obtain in-depth data on the views of the participant groups, in order to complement the quantitative data from the time-and-motion and survey sub-studies	1) Interviews and conversations with LHWs during the time-in-motion study, and impromptu conversations with clients during LHW visits were audio-recorded 2) Interviews with NGO staff and health authority managers 3) The recordings were transcribed and analysed thematically
	*Sub-study 5: Photo and voice diary keeping* by clients (Sites 1, 2)	To gain an in-depth understanding of how clients cope with their illness and the role that LHWs play in this. The diaries were trialed as an alternative to interviews	1) Participants received a disposable camera and/or audio recorder and we asked them to share how they coped with their illness/es, including their everyday interactions with LHWs and clinic staff, and the barriers and enablers to treatment adherence. These images and recordings were collected and discussed with the participant during monthly visits. Conversations were audio-recorded 2) The data were thematically analysed

The evaluation team comprised four researchers, who worked across the three study sites, over nine months. We conducted the time-and-motion study and the client survey within a specified time period (eight and nine weeks respectively). Data collection for the other sub-studies was done as and when participants were available. We developed protocols for each sub-study detailing the what, how, who and when of each evaluation activity. All instruments were piloted and refined before a sub-study was formally implemented (see Table 4 below for an example of a structured observation guide).

**Table 4:** The observation guide for LHW visits to clients

The evaluator wrote detailed notes of the visit, using the following headings:	
• Place where the visit took place	
• Visit time (time of the day and how long the visit lasted)	
• People present	
➢How many	
➢Apart from the client, who else, and what were their relationship to the client?	
• The conversation topics	
• The conversation pattern (who talked most; who introduced new topics; when were there silences?)	
• Apart from discussing the client’s health, what other service/s did the worker provide?	
• Any notable barriers for the LHW in delivering services?	
• How did the visit end?	

The data collection methods for the evaluation are shown in Table 5. Data from these sources were synthesised in the analysis to: (a) describe the models of care; and (b) explore the views and experiences of (i) clients; (ii) LHWs; and (iii) programme managers. Both the methods and divergent participant groups allowed us to identify similarities and, equally importantly, differences between the groups on how they perceived and experienced delivering healthcare services in the study sites.

**Table 5:** Participants in, and data collected for, each sub-study

Sub-study	Participants	Data collection
1. Time-and-motion	18 LHWs; 50% of the LHWs across the three sites including the four team leaders (Site 3 had two team leaders)	• The team leaders were observed whilst working in the clinic (26 hours) • The LHWs were accompanied on their treatment and adherence support visits (33 hours)
2. Client survey	226 clients (19% living with TB; 51% with HIV/AIDS; and 30% co-infected with TB and HIV) across the three study sites, as follows: • Site 1 = 31% (Female = 49%) • Site 2 = 31% (Female = 63%) • Site 3 = 38% (Female = 68%) All respondents were over 18 years of age	The questionnaire comprised clients’ assessment of: (i) LHW-visits (ii) TB and/or HIV counseling at the clinic (iii) Routine TB and/or HIV services at the clinic (iv) TB clients’ treatment location preference (v) Clients’ general knowledge of TB and HIV/AIDS
3. Structured observations	Four LHWs (all female) and seven clients	Five visits; two were with couples The observation times ranged between 30 to 60 minutes
4. Interviews	Two team leaders (both female); four LHWs (all female); two NGO managers (both female); two health facility staff (one male, one female); three health authority managers (all female)	Apart from the team leaders and LHWs, separate interview-schedules were drafted for each participant, given their different roles in the programme
5. Client diary keeping	Participant 1: Female, ± 40 years old, living with HIV/AIDS, audio-and visual diaries Participant 2: Male, 30 years old, on TB/HIV treatment, audio and visual diaries Participant 3: Female, between 30 and 36 years old, on TB/HIV treatment, audio-diary Participant 4: Male, 31 years old, living with HIV/AIDS, visual diary Participant 5: Male, 39 years old, living with HIV/AIDS, audio and visual diaries	The duration of participation ranged from four to nine months

## Results

We present below a critical assessment of each method’s challenges and strengths, and discuss how the data collected contributed to achieving the evaluation objectives. This is preceded by a brief summary of the overall evaluation findings.

### Summary of the evaluation findings

Overall, the evaluation [23] confirmed the feasibility of the programme, with its variations across the implementation sites, for providing integrated community-based treatment and adherence support to TB, HIV/AIDS and co-infected clients. The evaluation highlighted a number of strengths of the programme, in particular the dedicated LHW teams, and offered recommendations to address its challenges, such as the low proportion of LHWs’ time that was spent in direct contact with clients. Table 6 provides a summary of the key messages from the evaluation report.

**Table 6:** Key messages from the evaluation report (extracted from reference 23) http://www.mrc.ac.za/healthsystems/operationalresearch2010.pdf

1. The study confirms the feasibility of integrating community-based care for clients living with TB and HIV. Evaluation of the health outcomes of integrated models that are implemented at scale, and outside of research settings, is needed to confirm the effectiveness of these approaches.	
2. Providing support to co-infected clients using one LHW appears to be less intrusive and disruptive than having different LHWs support these clients, and is an important benefit of integrating community-based services.	
3. Clients were very positive about their experiences of services rendered by LHWs. The majority of clients on directly observed treatment for TB would prefer self-administered treatment at home, however a notable proportion of these clients indicated a preference for LHW support during self-administration.	
4. LHWs often become intimately involved in the psycho-social realities of clients and they noted that working with individuals with serious, and often stigmatised, diseases is emotionally stressful. It is therefore important that:	
a. LHW training include both the bio-medical aspects of TB and HIV and the psycho-social aspects of living with these diseases.	
b. ‘Caring for the Carer’ programmes be put in place to help LHWs manage these stresses.	
5. Identifying clients at-risk of non-adherence and who need intensified LHW care and support, and using this information to prioritise LHWs’ work, is an effective way to manage the caseload of LHWs. 6. Establishing and maintaining high morale among LHWs is an important component of ensuring the delivery of quality services. Providing non-monetary incentives in recognition of their work is as important to LHWs as increased stipends.	
7. The monitoring and evaluation tools used in the study sites strengthened the delivery of LHW services. These tools should be included in programmes that employ LHWs to provide treatment and adherence support to individuals living with TB and HIV.	

### Sub-study 1: Time-and-motion - describing and understanding the work of lay health workers and their team leaders

The time-and-motion study, reported in full elsewhere [29], had several strengths. First, it complemented the data obtained from interviews with the LHWs. The observations helped to address our concerns that (i) the interview data may have been affected by poor recall of earlier events (since the interviews were conducted towards the end of the data collection period), and (ii) LHWs and team leaders may have emphasised views and activities that they saw as reflecting favourably on them.

Second, the data highlighted the impact of context on programme implementation. For example, the findings showed that, on average, LHWs spent only 46% of their time with clients. The remainder of their time was spent on walking to, and waiting at, clients’ houses – a reflection of the large geographical areas they covered and the challenge of making appointments with clients who could only be contacted in person. This information is important to understanding what might constitute realistic work targets for community-based LHWs, allowing programme managers to plan appropriately during programme implementation.

Third, the observations identified a number of innovative practices that may otherwise not have been revealed. For instance, knowing that TB and HIV/AIDS are highly stigmatised in the communities in which they worked, LHWs developed a range of strategies to protect the confidentiality of their clients. In one case, the LHW pretended to be ‘just a friend visiting’ when people who were not aware of the HIV positive status of the client arrived unexpectedly. In another case, the LHW concealed her role from other community members by placing her work papers inside a popular magazine. Both strategies reduced the likelihood of accidental disclosure of the client’s status within the neighbourhood.

Fourth, this sub-study provided us with unanticipated opportunities to engage the team leaders and LHWs in spontaneous, informal conversations. The data from these conversations contributed substantively to our understanding of the day-to-day realities of the work of LHWs. For example, during one set of observations a LHW casually showed us where a recent gang-related shooting had happened. This led to a conversation about work-related risks, but also revealed LHWs’ perception that they were treated with respect in the communities in which they worked and were seldom the target of crime and gang-related violence. These data, in turn, led to insights regarding the importance that LHWs attach to being respected and appreciated by the communities in which they work and by their managers.

Finally, the contact with clients during these observations provided opportunities to probe emerging issues. For instance, one encounter raised the question of whether age differences between LHWs and clients impact on their relationship and, if so, how. These unscripted, spontaneous interviews yielded such rich data that we recorded these for all nine time-and-motion participants in Sites 2 and 3 (but not in Site 1 as the data collection was already completed).

There were, however, two important challenges in implementing this approach. Firstly, it was labour intensive and therefore only feasible to implement with a small sample of LHWs over a limited time period. Ideally, this sub-study would have run over a longer time period to ascertain if other factors, such as seasonal changes, impact on LHW activities. Secondly, some LHWs may have wanted to emphasise their challenging working conditions and may therefore have chosen, on time-and-motion observation days, to visit problematic clients who lived furthest from the clinic that was responsible for their care. However, all of the clients visited had been assigned to the LHWs at the time of the evaluation, and it is therefore likely that the data provided a reasonable reflection of LHWs’ daily work.

### Sub-study 2: Survey - clients’ assessment of the programme components

A key strength of the client survey was that it highlighted important implementation differences across the study sites. For example, 92% of client participants in Site 1 reported having received weekly or bi-weekly LHW visits, compared to the 36% and 52% of participants in Sites 2 and 3 respectively. The information from participants also indicated that some LHWs were not adhering to visit guidelines developed by the managing NGO: only 51 (36%) of the 142 clients on ART, or on ART and TB treatment, reported that they had received adherence support visits. The visit guidelines note that all ART clients should be visited regularly.

These results were met with concern by the NGO who managed the LHWs and commissioned the evaluation. The NGO asked us to check our analysis and to conduct focus group discussions (FGDs) with LHWs to confirm the survey findings. This further work did not change our findings, but provided useful additional data on how context shapes programme implementation: in the FGDs, LHWs with high client caseloads explained that it made sense to prioritise visits to clients who were not doing well on treatment. Those who were doing well, they noted, were not visited unless this was requested by the client or their health facility. This illustrates the utility of the relatively simple survey that we conducted in identifying potentially important implementation issues that required further exploration using other methods. These additional data, in turn, contributed to generating a more nuanced understanding of how LHWs organise their work.

### Sub-study 3: Structured observations - describing the ‘intervention moments’ of the programme

The observations of LHWs’ interactions with clients, which we defined as the intervention moments of the programme, were far more challenging than the time-and-motion observations because of their intention to involve both clients and LHWs. The challenges we encountered included clients being ill at ease with the presence of the observer; clients appearing to report only positive aspects of the care they received from LHWs; clients not reporting their non-adherence to treatment; and LHWs pressuring the observer to help with clients who they viewed as problematic. These suggest important power imbalances, not only between the research observer and clients but also between LHWs and their clients.

Our observations of these intervention moments helped to confirm the findings of the time-and-motion sub-study regarding the high levels of stigma attached to TB and HIV/AIDS in these communities. For example, they revealed how LHWs used nonverbal approaches to communicate with clients regarding an impending visit, in order to protect their status from other people. These additional data therefore offered insights into issues that shaped programme delivery and that may otherwise not have come to our attention.

An unintended consequence of the sub-study was the close relationship that developed between the observer and the LHWs whom she shadowed, allowing the LHWs to more easily share their experiences. For example, LHWs discussed during the observations that they felt that their training did not equip them sufficiently to work with and support clients who were not taking their treatment as prescribed. Another unexpected consequence of the observational work was that the LHWs asked the observer to help them with clients who were not adhering to their treatment.

### Sub-study 4: Interviews -exploring the views and experiences of clients, LHWs, NGO staff and managers and health authority managers

These formal, structured interviews were intentionally conducted towards the end of the evaluation. We drew on the preliminary findings from the time-and-motion and structured observation studies, as well as the preliminary survey results, to develop interview schedules. These were therefore informed by the on-the-ground realities of the programme and helped us to clarify issues raised by other components of the evaluation, in particular the reasons for differences in the way the programme was implemented across the study sites. For instance, we found after probing that the health authorities had sanctioned variations in LHW visit protocols, as they had not finalised and standardised a model of care. Another example of how the interviews helped to clarify questions emerging from other data sources concerned our time-and-motion observation that team leaders often felt overwhelmed by their duties. When we raised this with them during the interviews, they explained that they found it particularly stressful to deal with instances in which LHWs were not adhering to visit or other guidelines and, at the same time, maintain a high morale amongst their LHW team.

### Sub-study 5: Diary keeping - exploring how clients coped with their illness and the roles that LHWs played in this

The diary data provided fascinating information about the day-to-day struggles of clients to cope with their illness in the context of extreme poverty and appalling living conditions. A key strength of this method was the unique insights it provided into the ways in which LHWs support clients, which went as far as one LHW visiting a client in prison. The study also offered longitudinal data on the treatment trajectory for two people living with HIV who were initiated on ART shortly before being recruited for this sub-study. Diary keeping allowed us to understand the high value placed by clients on receiving support from someone who was familiar with their living conditions and circumstances, and who understood the challenges that they faced.

Although the diary keeping provided valuable data, it is a labour intensive approach and needs to be continued over long periods of time if the illness experiences of people are to be captured adequately. In this evaluation, two participants kept diaries for 24 weeks while another two participants were involved for the full nine months of the evaluation. The volume of data generated over these long periods is challenging to analyse. An additional challenge lies in the recruitment of participants: not all people have the capacity to keep a diary or interest in doing so, and careful explanation and recruitment is therefore needed. In our study we asked the LHWs to identify potential ‘diary participants’. Given these challenges, diary-keeping may not be a useful tool for all formative evaluations. Evaluators need to weigh the benefits of the very in-depth data obtained against the resources needed to collect and analyse these.

## Discussion

We reflect below on the methodological concerns cutting across the methods used in this evaluation and attempt to identify important issues in planning and conducting formative evaluations of complex interventions using multiple, mixed-methods. Based on these reflections, we also make suggestions for future evaluations (Table 7).

**Table 7:** Suggestions for optimising the benefits of multiple, mixed-method formative evaluations

• Multiple, mixed method formative evaluations require careful planning to select appropriate methods, develop appropriate data collection instruments, sequence data collection, collect data and undertake analysis in ways that both does justice to the individual methods and allows data to be triangulated across methods	
• The evaluation protocol should include information on the methods and approaches that will be used to triangulate, and in some cases integrate, the findings from each of the evaluation methods used	
• Consultation with and involvement of key stakeholders, including those commissioning the evaluation, can help to ensure that appropriate methods are selected to address the evaluation questions	
• The evaluation plan should include opportunities for the evaluation team to reflect on whether the methods selected are achieving their objectives and whether changes need to be made to the mix of methods selected or their sequencing within the overall evaluation	
• Multiple methods can easily overstretch the resources of the evaluation team. A judicious balance needs to be struck between what is practically feasible, in terms of resources, time and the skills of the evaluation team; what is needed to address the evaluation questions; and what is needed to ensure the scientific rigour of the evaluation	
• Careful planning and continuous reflection are needed when trying out innovative methods not used previously	
• Opportunities to feed findings back to stakeholders need to be built into the evaluation plan. Ideally, these should include opportunities during the evaluation process, for example when preliminary results from each method are available, and at the end of the evaluation, to obtain input on the integrated findings	

### Multiple, mixed-methods generate a more detailed and textured evaluation

It has been suggested that neither the number of methods, nor the mix of qualitative and quantitative methods, determine the quality of an evaluation, but rather the extent to which these align with the questions the evaluator seeks to answer [7]. The first objective of this evaluation was to provide a detailed description of the programme and the second was to explore the perspectives and experiences of all the social actors involved in it. We anticipated that one method would not easily achieve these objectives and therefore chose a multi-method approach which we hoped would result in more comprehensive and holistic evaluation findings. This holistic approach is illustrated by our findings on team leaders’ work, and their perceptions and experiences of this. The time-and-motion study provided a quantitative description of how they organised their time while the interviews added insights into their feelings of being overburdened with work demands. This triangulation involved exploring and comparing data on the same phenomenon, in this case what it meant to be a team leader, that we had collected using different methods [11, 16]. It offered a more inclusive and textured account than would have been possible using only one data collection method. We would also argue that the triangulation of data from different methods also strengthened the trustworthiness of the data [11, 13, 16].

Similarly, the diary keeping and observations yielded rich data regarding the life and health experiences of people living with stigmatised illnesses and attempting to manage complex treatment programmes under very difficult conditions. These two sub-studies gave us insights into the “hidden moments” of community-based healthcare. One such moment was observing the touching empathy and support with which a LHW comforted and encouraged a client with HIV and AIDS who wanted to give up on life. This observation prompted us to look more carefully at the commitment that LHWs showed to their clients and the reasons for this. We were also able to triangulate the findings from the diary keeping and observations with the survey findings which also focused on clients’ experiences of the healthcare services they received. For example, the survey showed that 92% of clients felt that the LHW visits were helpful or very helpful. This perception was repeatedly echoed by clients during the diary keeping and structured observations of LHW visits.

The concept of triangulation is used widely within the mixed method evaluation literature, and focused initially on the idea that similar results from different research methods would enhance the validity of research findings [18]. Subsequently, triangulation has also been conceptualised as a mechanism for producing a more complete or holistic picture of the phenomenon of interest [18]. Denzin [19] identified a range of different triangulation strategies: data triangulation, using multiple sources of data; investigator triangulation, using varied observers; and theoretical triangulation, in which theories from multiple disciplines are used to broaden the interpretive framework of a study [19, 31]. While these different forms of triangulation are often promoted, it has been argued that insufficient attention has been paid to what triangulation means both epistemologically and at the level of methods, interpretation, and inference for individual studies [18, 31]. Further, triangulation is only one of a range of reasons for combining different methods – other reasons include complementarity (using results from one method to explain results from another), development (using results from one method to further the development of another) and expansion (using different methods for different elements of the research question so as to expand its depth) [32]. While this ‘fuzziness’ is conceptually challenging, Erzberger and Kelle note that the different approaches to triangulation are helpful in identifying different options for drawing together results from different methods and that no single approach is appropriate for all types of integration [18].

When planning the evaluation, we believed that the mix of quantitative and qualitative methods selected and their triangulation would complement each other [33], provide a balance between breadth of data (a strength of the client survey) and depth of data (a strength of the client diaries and structured observations), and allow us to expand the depth of the research overall. Together these methods would also contribute to the generalisability of the findings [34, 35]. In practice, we found that this approach did help us to distill key implementation findings and principles [7]. For example, the evaluation identified the need for the training of LHWs to include both bio-medical knowledge – a conclusion drawn from findings from the client survey – and ways of managing psychosocial barriers to treatment adherence – a conclusion derived from the results of the structured observations and diary-keeping. However, the range of methods that we selected and our approach to both data and investigator triangulation raised a number of challenges which we discuss below.

### A limit to the multitude of methods?

Enthusiasm for multiple methods can easily turn into an overly ambitious evaluation that over-stretches its resources. Multi-method evaluations need considerable resources to collect, analyse and report data and also require that the research team include a wide range of skills and experience [20, 21]. Our research team had worked together for some time and were committed to the mixed methods paradigm [36]. However, bringing together researchers from different methodological paradigms may be challenging for both team processes and data analysis [36]. Our experience suggests that careful planning and agreement on the value and combination of methods is needed before embarking on an evaluation.

We also need to recognise that more effort is needed to plan and conduct mixed methods evaluations, compared to single method evaluations. Qualitative and quantitative research make different epistemological assumptions, and researchers have questioned whether the interpretivist and positivist paradigms are compatible at all [37], and the implications of this for evaluations that attempt to bring together qualitative and quantitative data. In this study, we took a pragmatic stance [9, 35, 38]: we accept that there are multiple realities that can be explored empirically and focused on understanding practical problems identified by our colleagues in practice settings, with the aim of providing useful knowledge. Guided by this approach, we saw it as important that the methods we used were complementary and helped to achieve the evaluation objectives, while taking available resources and time constraints into account. An evaluation with an array of sophisticated and sensitive data collection tools, but without adequate time and resources to implement these appropriately or to do justice to the emerging data, is unlikely to produce valid and trustworthy findings and may be less desirable than a well conducted single method evaluation. For example, the rich, in-depth longitudinal data gathered through methods such as diary keeping need to be traded off against the time needed to collect and analyse these data. Diary keeping may be most useful for issues that are sensitive or stigmatised, and have profound effects on people’s daily lives. The in-depth data produced through this method can help understand changes in people’s views and experiences over time. Diary keeping could also be considered for formative evaluations of novel interventions whose potential impacts are not well understood. However, it may not produce useful knowledge where research resources are very limited or where rapid results are needed.

One of the most challenging aspects of multi-method evaluation is the analysis of data from different methods and sources [38]. Ideally, this process is iterative with preliminary findings and methodological insights from one method informing the application of the other methods. Data analysis needs to explore the results from each approach while making connections between these emerging groups of results, with a view to developing an integrated set of findings. Methods designed for applied research, such as framework analysis, may offer advantages as these allow data to be categorised and analysed in a structured manner [35, 39, 40]. Logic models are another analytic tool that can be helpful in drawing together findings from different methods to create hypotheses regarding how a process unfolds or how an intervention impacts on its intended recipients and is influenced by its delivery and wider context [41, 42].

An important practical consideration is whether there is sufficient time to provide timely and regular feedback to the commissioning agency and other stakeholders during the course of the evaluation [43, 44]. In our evaluation, the timeframe required by the funder meant that feedback of preliminary findings from each evaluation component was not feasible and only the findings from the client-survey were shared with stakeholders in a feedback meeting. All other results were jointly presented to stakeholders one month after completion of the evaluation. We therefore did not have opportunities to discuss all preliminary findings – a process that could have been useful in understanding each set of findings and also in making connections across the different sets of findings. While mixed method formative research can forefront the voices of different stakeholders, including service users and providers [38, 45], our experience raises questions of how and when to best engage with stakeholders regarding the findings of mixed method evaluations, so as to be able to draw in their perspectives, and how to present rich and textured mixed method findings to these groups and to other researchers.

### Evaluation as a creative and innovative enterprise

Patton argues that programme evaluation is as much a creative process as it is rooted in sound and robust methodologies [7]. It is this perspective that led us to experiment with less frequently used evaluation methods such as structured observations and diary keeping. We learnt valuable lessons through this experimentation – for example, we did not anticipate that the presence of the observer, who had the same ethnic and language background as the clients and LHWs, would affect clients to the extent that we felt their responses during the structured observations were partial or overly positive. In future evaluations we need to consider alternative strategies to address this problem. Similarly, we found the large volumes of data produced by diary keeping difficult to manage in a time-limited evaluation. However, we believe that the mix of methods led to useful knowledge overall – a key feature of the pragmatic stance to mixed method research [9] – and we learnt a number of valuable lessons, including on the many challenges of evaluating a community-based programme to support people living with stigmatised illnesses. An innovative and creative evaluation allows the evaluation team to be responsive to data collection opportunities in the field, such as our decision to record informal conversations during the time-and-motion study – a data collection element that was not planned initially. These changes of course need to be congruent with the evaluation protocol and ethics approvals.

New and innovative methods place the evaluation team on unfamiliar ground regarding what these methods will entail. For example, the diary-keeping fieldwork became emotionally stressful for the evaluator because of the intimate information shared by the participants. This highlights the importance of reflexivity and of support for both participants and evaluators. While empathy and immersion are important considerations when researching people’s experiences of living with a health issue, and close relationships with participants and communities can help to obtain a holistic view of the programme evaluated, care should be taken to ensure that appropriate debriefing opportunities are available.

### Suggestions for optimising the benefits of multiple, mixed-method evaluations

From the experiences described in this paper, we have distilled a number of suggestions that may help other researchers optimise the benefits of multiple, mixed-method evaluations (Table 7).

## Conclusions

Formative evaluations are not a ‘miracle cure’ that offer definitive answers to programme implementation questions but rather their value lies in their iterative nature. In the evaluation discussed here, each sub-study, individually and combined, contributed to opening up the ‘black box’ of how and why this programme functioned as it did. Based on the lessons we learned from this evaluation, the answer to the question posed in the title is a definite ‘Yes’ – formative evaluation designs including multiple qualitative and quantitative methods hold distinct advantages over single method evaluations. However, their value is not in the number of methods used, but in how each method matches the evaluation questions.

## Abbreviations

ART: Antiretroviral therapy
DOT: Directly observed therapy
FGD: Focus group discussion
LHWs: Lay health workers
NGO: Non-governmental organisation
